# Second Primary Malignancies in Adults with Gastric Cancer – A US Population-Based Study

**DOI:** 10.3389/fonc.2016.00082

**Published:** 2016-04-11

**Authors:** Binay Kumar Shah, Amit Khanal, Yvonne Hewett

**Affiliations:** ^1^PeaceHealth United General Medical Center, Lewiston, ID, USA; ^2^Section of Hematology and Oncology, Department of Medicine, University of Illinois at Chicago, Chicago, IL, USA

**Keywords:** gastric cancer, second primary malignancy, SEER, latency

## Abstract

**Background:**

Multiple studies have examined the incidence of secondary primary malignancies (SPMs) in gastric cancer patients in Europe and Asia. This retrospective review was conducted to analyze risk of SPM in patients with gastric cancer diagnosed in the United States.

**Methods:**

We included adult patients diagnosed with gastric cancer from the surveillance, epidemiology, and end result (SEER) 13 database. We calculated the risk of SPMs in these patients using the multiple primary standardized incidence ratio session of SEER*stat software and performed subset analyses of SPM with regard to age, sex, radiotherapy used, and latency period.

**Results:**

Among 33,720 patients, 1838 (5.45%) developed 2019 SPMs with an observed/expected (O/E) ratio of 1.11 [95% confidence interval (CI) = 1.06–1.16, *p* < 0.001] and an absolute excess risk of 18.16 per 10,000 population. The median time to first SPM from the time of diagnosis of gastric cancer was 46.9 months (range 6–239 months). Significant excess risk was observed for gastrointestinal malignancies [O/E ratio 1.71 (CI = 1.59–1.84, *p* < 0.001)], thyroid [O/E ratio 2.00 (CI = 1.37–2.8, *p* < 0.001)], and pancreatic cancer [O/E ratio 1.60 (CI = 1.29–21.96, *p* < 0.001)]. Risk of secondary melanoma, breast cancer, and prostate cancer was lower than in the general population.

**Conclusion:**

The risk for SPMs is significantly increased in adults with gastric cancer compared to the general population.

## Introduction

Gastric cancer is the fifth most common cancer and the third leading cause of cancer-related death worldwide. The highest incidences are found in Eastern Asia and Central and Eastern Europe (1). Although the incidence of gastric cancer has declined over the last half century, the prognosis remains poor (2).

The majority of patients in the United States (US) present with regional or distant metastases, with an overall 5-year survival rate of 28.3% (1). By contrast, most Japanese gastric cancer patients are diagnosed in early stages of the disease and have an overall 5-year relative survival rate of over 50% (3).

Geographic disparities exist in treatment modality as well (4). Surgery remains the only potentially curative option (5). Although multimodal treatment is the standard of care for bulky localized disease, adjuvant chemotherapy is typically employed in Asia, adjuvant chemoradiation in North America, and neoadjuvant chemotherapy in European and Australasian nations (4). Patients with advanced or recurrent disease typically receive systemic chemotherapy, as it provides a survival advantage over other treatments (6).

Gastric cancer survivors are at risk for secondary primary malignancies (SPMs). This long-term complication was first described by Warren and Gates in 1932 (7). Synchronous malignancies are defined as occurring with 6 months of the primary diagnosis, whereas metachronous tumors occur more than 6 months after the initial event. The risk of SPM is dependent on factors including patient age, cancer stage at diagnosis, and treatment modality (8).

The risk of SPM in gastric cancer patients in the US is unknown. Given the global variations in gastric cancer incidence, treatment, and survival, we evaluated the risk of SPMs in adult gastric cancer patients in the US to determine if geographic disparities are present. In addition, increased awareness of SPMs will provide the framework for pilot intervention studies and the development of evidence-based guidelines for gastric cancer survivors.

## Methodology

### About SEER Database

These data represent 14% of the US population, including the geographical areas of Atlanta, Connecticut, Detroit, Hawaii, Iowa, Los Angeles, New Mexico, rural Georgia, San Francisco-Oakland, San Jose-Monterey, Seattle (Puget Sound region), Utah, and Alaska Natives (1). Comprehensive cancer data, including primary tumor site, staging, patient demographics, treatment, and survival from hospitals and cancer treatment facilities, are subjected to rigorous quality control studies, resulting in a mature database, with 98% case completeness.

### Study Population

We selected adult patients aged 18 years or more, diagnosed with gastric cancer as first primary malignancy from January 1992 to December 2012. Patients were followed from diagnosis to the date of last known vital status, death, or the last point of data collection. We excluded cases diagnosed at autopsy and those lost to follow-up. Using the Warren and Gates criteria (7) as modified by the NCI (9), SPM was defined as metachronous malignancy developing 6 months or more after an index gastric carcinoma.

### Data Analysis

We used SEER*stat software, Version 8.2.1 April 8, 2015 to calculate the standardized incidence ratio (SIR), absolute excess risk (AER), and confidence interval (CI) for SPM in patients, as previously diagnosed with gastric cancer. We analyzed that the risks of SPM by latency period (6–23 versus 24 months or more) and exposure to radiotherapy were determined.

The SIR, which is also known as the relative risk, is a relative measure of the strength of association between two cancers. It is calculated by dividing the observed incidence of SPM by the expected incidence of SPM [observed/expected (O/E) ratio] in the general population. AER is an absolute measure of the clinical burden of additional cancer per 10,000 occurrences in the population. It measures the actual number of excess events normalized to the number of person years observed [AER = (O − E) PY].

## Results

A total of 33,720 patients diagnosed with primary gastric cancer met the study criteria. Patient demographics are presented in Table 1. A total of 1838 patients (5.26%) developed 2019 SPMs with an O/E ratio of 1.11 (CI = 1.06–1.16, *p* < 0.001; AER 18.16) (Table 2). A total of 1676 patients developed 1 SPM, and 162 developed 2 or more SPMs. The average age at diagnosis of SPM was 74 years (24–104 years). The median time from primary diagnosis to development of first SPM was 46.9 months (range 6–239 months). Significant excess risk was observed for gastrointestinal, biliary, pancreatic, and thyroid cancers. Significantly, decreased risk was observed for secondary melanoma, breast, and prostate cancer.

**Table 1:** **Demographics of patients**.

	Number (%)/median (range)
Total number of patient with gastric cancer	33,720
Gender	
Male	20,474 (60.72%)
Female	13,246 (%)
Race	
White	22,451 (66.58%)
Black	3942 (11.69%)
Other	7327 (21.73%)
Total number of SPM	2019
Total number of patient with SPM	1838 (5.45% of study population)
Total number of patient with 1 SPM	1676 (4.97% of study population)
Gender	
Male	1084 (64.68%)
Female	592 (35.32%)
Race	
White	1126 (67.18%)
Black	210 (12.53%)
Other	340 (20.29%)
Total number of patient with 2 or more SPM	162
Gender	
Male	115 (70.99%)
Female	47 (29.01%)
Race	
White	112 (69.14%)
Black	22 (13.58%)
Other	28 (17.28%)
Age at the time of diagnosis of SPM (median)	74 years (24.3 years–104.5 years)
Latency to develop first SPM	3.91 years (6 months–19.91 years)
Follow-up time (median)	6.91 years (6 months–20.91 years)

**Table 2:** **Total SPM**.

	Total				
	Person at risk = 33,720; Person year at risk = 107,690.38				
	*N*	O/E	Two tail *p*	Confidence interval	Excess risk
All sites	2019	1.11	<0.001	1.06–1.16	18.16
All sites excluding non-melanoma skin	2011	1.11	<0.001	1.06–1.16	18.16
All solid tumors	1813	1.12	<0.001	1.07–1.18	18.54
Brain and other CNS tumor	12	0.74	0.349	0.38–1.29	−0.4
Head and neck	36	0.62	0.002	0.43–0.85	−2.07
Thyroid	33	2	<0.001	1.37–2.8	1.53
Lung and bronchus	299	1.09	0.153	0.97–1.22	2.26
Esophagus	51	2.41	<0.001	1.79–3.17	2.77
Stomach	217	4.51	<0.001	3.93–5.16	15.69
Small intestine	24	3.06	<0.001	1.96–4.55	1.5
Colon, rectum, and anus	254	1.15	0.03	1.01–1.3	3.11
Liver, gallbladder, and other biliary	69	1.3	0.04	1.01–1.65	1.48
Pancreas	92	1.6	<0.001	1.29–1.96	3.19
Breast	124	0.8	0.011	0.67–0.95	−2.89
Female genital system	64	1.07	0.615	0.82–1.37	0.4
Male genital system	300	0.79	<0.001	0.7–0.88	−7.41
Urinary system	182	1.11	0.173	0.95–1.28	1.68
Soft tissue including heart	10	1.05	0.964	0.5–1.93	0.04
Skin excluding basal and squamous	40	0.64	0.003	0.46–0.87	−2.09
Lymphoma	76	0.95	0.709	0.75–1.19	−0.37
Myeloma	27	0.97	0.97	0.64–1.41	−0.08
Acute lymphocytic leukemia	3	2.08	0.352	0.43–6.08	0.14
Chronic lymphocytic leukemia	15	0.73	0.261	0.41–1.21	−0.51
Acute myeloid leukemia	22	1.44	0.122	0.9–2.18	0.63
Chronic myeloid leukemia	11	1.71	0.126	0.85–3.06	0.42
Kaposi sarcoma	3	2.06	0.357	0.43–6.03	0.14

### SPM and Age

All patients had an increased risk of gastrointestinal second primary malignancies. On subgroup analysis, older patients had a significantly higher risk of SPMs of the esophagus (O/E ratio of 2.28, CI = 1.67–3.06, *p* < 0.0001; AER 3.15), stomach (O/E ratio of 3.66, CI = 3.13–4.26, *p* < 0.0001; AER 15.11), small intestine (O/E ratio of 2.93, CI = 1.81–4.48, *p* < 0.0001; AER 1.72), thyroid (O/E ratio of 1.95, CI = 1.25–2.9, *p* < 0.005; AER 1.45), and pancreas (O/E ratio of 1.55, CI = 1.24–1.92, *p* < 0.001; AER 3.77). Younger patients were at increased risk of malignancies of lung and bronchus (O/E ratio of 2.05, CI = 1.36–2.96, *p* < 0.001; AER 5.24), esophagus (O/E ratio of 4.05, CI = 1.49–8.81, *p* < 0.001; AER 1.65), stomach (O/E ratio of 20.56, CI = 15.26–27.11, *p* < 0.0001; AER 17.39), colon (O/E ratio of 2.91, CI = 2.09–3.95, *p* < 0.0001; AER 9.84), and urinary system (O/E ratio of 2.47, CI = 1.57–3.71, *p* < 0.001; AER 5).

### SPM and Latency

The median time from primary diagnosis to development of first SPM was 47 months (range 6–229 months). All solid tumors were significantly increased after 24 months of latency (O/E ratio of 1.18, CI = 1.12–1.24, *p* < 0.001; AER 30.42). The risk of malignancies of thyroid (O/E ratio of 3.17, CI = 1.77–5.22, *p* < 0.001; AER 3.08), esophagus (O/E ratio of 2.52, CI = 1.44–4.09, *p* < 0.005; AER 2.89), and small intestine (O/E ratio of 4.03, CI = 1.84–7.64, *p* < 0.005; AER 2.03) was significantly increased within 6–23 months of the index diagnosis. The risk of malignancies of esophagus (O/E ratio of 2.36, CI = 1.64–3.28, *p* < 0.0001; AER 2.71), stomach (O/E ratio of 5.34, CI = 4.59–6.17, *p* < 0.0001; AER 19.9), small intestine (O/E ratio of 2.67, CI = 1.49–4.4, *p* < 0.005; AER 1.26), hepatobiliary system (O/E ratio of 1.54, CI = 1.17–1.99, *p* < 0.005; AER 2.79), pancreas (O/E ratio of 1.69, CI = 1.32–2.13, *p* < 0.0001; AER 3.84), and lung and bronchus (O/E ratio of 1.18, CI = 1.03–1.34, *p* < 0.05; AER 4.69) was increased after 24 months of latency compared to the general population.

### SPM and Radiation Exposure

Of the 1838 patients who developed SPM, 421 received radiotherapy. The risk of SPM at all sites was significant in both groups. Among patients with no radiation exposure, the risk of SPMs of the thyroid (O/E ratio of 1.69, CI = 1.06–2.56, *p* < 0.05; AER 1.06), esophagus (O/E ratio of 2.1, CI = 1.46–2.92, *p* < 0.0001; AER 2.16), stomach (O/E ratio of 4.43, CI = 3.8–5.13, *p* < 0.0001; AER 16.04), small intestine (O/E ratio of 3.17, CI = 1.94–4.9, *p* < 0.0001; AER 1.61), and pancreas (O/E ratio of 1.52, CI = 1.19–1.91, *p* < 0.005; AER 2.9) was significantly increased. Patients with exposure to radiation had increased the risk of thyroid (O/E ratio of 3.13, CI = 1.56–5.6, *p* < 0.005; AER 3.3), esophagus (O/E ratio of 3.57, CI = 2.04–5.8, *p* < 0.0001; AER 5.08), stomach (O/E ratio of 4.96, CI = 3.56–6.73, *p* < 0.0001; AER 14.44), large bowel (O/E ratio of 1.52, CI = 1.16–1.96, *p* < 0.005; AER 8.92), and pancreas (O/E ratio of 1.96, CI = 1.2–3.02, *p* < 0.01; AER 4.31).

## Discussion

There are currently more than 13 million cancer survivors in the US, with a projected increase to 18 million by 2022 (10). Early diagnosis and development of new agents continue to improve cancer outcomes. Long-term morbidity and premature mortality may occur in survivors, as a result of comorbidities, treatment, or disease-related factors.

In 2006, the Institute of Medicine report recommended the use of evidence-based guidelines to identify and manage the long-term effects of cancer, including surveillance for and prevention of recurrences and SPMs (11). Studies such as this not only increase awareness of secondary malignancies but also provide valuable information that may be used to pilot interventions in care leading to improved outcomes for cancer survivors. SPMs are common in cancer patients with an overall cumulative incidence of 14% at 25 years of follow-up (12).

Previously published literature has examined the incidence of SPMs in gastric cancer patients in Asia, Europe, and the Middle East. This is the first study to examine the incidence of SPM in gastric cancer patients in the US. Previous studies are inconsistent regarding the risks for SPM in gastric cancer, but our results are in agreement with the literature that suggests an increased risk for colon, liver, and pancreatic cancers (13–17). Many gastric cancer patients may receive radiotherapy, a known risk factor for secondary malignancies. A recent cohort study estimated the excess risk at 8% in patients surviving more than 1 year following treatment, resulting in 5 SPMs per 1000 radiotherapy patients by year 15 after diagnosis (18). We have summarized rates of SPMs reported in the literature (Table 3). Our study is the largest study in the last 25 years. Contrary to the study by Hiyama et al., our study showed significant increase in risk of SPM in gastric cancer patients. Findings of our study will help us formulate evidence-based guidelines for follow-up of gastric cancer survivors.

**Table 3:** **Second primary malignancies in gastric cancer**.

Reference	Number of patients	Number of SPM	Odd’s ratio (95% CI)
Hiyama et al. (14)	61,168	1045	M: 0.84 (0.78–0.90)
			F: 0.90 (0.79–1.01)
Lundegardh et al. (15)	34,506	962	1.16 (1.09–1.24)
Ikeda et al. (16)	2250	47	N/A
Bozzetti et al. (13)	105	10	N/A
Ikeda et al. (17)	1070	54	N/A
Current study	33,720	2019	1.11 (1.06–1.16)

*N/A, not available; SPM, second primary malignancies; M, male; F, female; CI, confidence interval*.

The strengths of this study include its large sample size and long-term follow-up. The limitations of the study are related to the use of population-based registries. Surveillance, epidemiology, and end result (SEER) database does not have comorbidities, lifestyle and risk factors, environmental exposure, and family history. SEER database does not have information on chemotherapy used. We were unable to analyze SPM by stage, because collaborative stage is not used for cases diagnosed prior to 2004 (19).

The risk of SPM is significantly increased in US patients with gastric cancer. The risk of any specific SPM is dependent on treatment and time of latency. Evaluation for SPMs is critical in the follow-up of gastric cancer survivors.

## Author Contributions

BS: concept/hypothesis, data acquisition, interpretation, and analysis; drafting of manuscript. AK and YH: data analysis and interpretation, critical revision for intellectual content; drafting of manuscript.

## Author Note

This paper was presented as a poster at Gastrointestinal Cancers Symposium, January 15–17, 2015 at San Francisco.

## Conflict of Interest Statement

The authors declare that the research was conducted in the absence of any commercial or financial relationships that could be construed as a potential conflict of interest.
